# Minutes of the Twenty-fourth and Twenty-fifth Annual Meetings of the State Medical Society of Kentucky

**Published:** 1880-11

**Authors:** 


"MINUTES OF THE TWENTY-FOURTH AND TWENTY-FIFTH ANNUAL MEETINGS OF THE STATE MEDICAL SOCIETY OF KENTUCKY.—The sensible plan adopted by this society, furnishes a worthy example for *all* State Societies. Under this arrangement the author of the paper is at liberty to publish the same *wherever*, however, and whenever he pleases. Bad and indifferent papers will therefore come to an end in the making and reading; useful papers will speedily find their way into the columns of medical journals, where they will be much more widely circulated than if buried in the chaff of the transactions such as a State Medical Society generally has reason to be ashamed of." *Chicago Medical Review, Oct. 20th, 1880.*

